# *Rana chensinensis* Ovum Oil Based on CO_2_ Supercritical Fluid Extraction: Response Surface Methodology Optimization and Unsaturated Fatty Acid Ingredient Analysis

**DOI:** 10.3390/molecules25184170

**Published:** 2020-09-11

**Authors:** Yuanshuai Gan, Dongliang Xu, Jianqiu Zhang, Zhongyao Wang, Shihan Wang, Hongye Guo, Kexin Zhang, Yajing Li, Yongsheng Wang

**Affiliations:** 1 School of Pharmaceutical Sciences, Jilin University, Changchun, Jilin 130021, China; ganys18@mails.jlu.edu.cn (Y.G.); xudl19@mails.jlu.edu.cn (D.X.); jqzhang19@mails.jlu.edu.cn (J.Z.); zhongyao18@mails.jlu.edu.cn (Z.W.); guohy18@mails.jlu.edu.cn (H.G.); zhangkx2818@mails.jlu.edu.cn (K.Z.); liyj2818@mails.jlu.edu.cn (Y.L.); 2 College of Chinese Medicine Materials, Jilin Agricultural University, Changchun, Jilin 130118, China; wsh8805@163.com

**Keywords:** supercritical fluid extraction, by-product, *Rana chensinensis* ovum oil, design of experiment, response surface methodology, Box–Behnken design, unsaturated fatty acids

## Abstract

*Rana chensinensis* ovum oil (RCOO) is an emerging source of unsaturated fatty acids (UFAs), but it is lacking in green and efficient extraction methods. In this work, using the response surface strategy, we developed a green and efficient CO_2_ supercritical fluid extraction (CO_2_-SFE) technology for RCOO. The response surface methodology (RSM), based on the Box–Behnken Design (BBD), was used to investigate the influence of four independent factors (pressure, flow, temperature, and time) on the yield of RCOO in the CO_2_-SFE process, and UPLC-ESI-Q-TOP-MS and HPLC were used to identify and analyze the principal UFA components of RCOO. According to the BBD response surface model, the optimal CO_2_-SFE condition of RCOO was pressure 29 MPa, flow 82 L/h, temperature 50 °C, and time 132 min, and the corresponding predicted optimal yield was 13.61%. The actual optimal yield obtained from the model verification was 13.29 ± 0.37%, and the average error with the predicted value was 0.38 ± 0.27%. The six principal UFAs identified in RCOO included eicosapentaenoic acid (EPA), α-linolenic acid (ALA), docosahexaenoic acid (DHA), arachidonic acid (ARA), linoleic acid (LA), and oleic acid (OA), which were important biologically active ingredients in RCOO. Pearson correlation analysis showed that the yield of these UFAs was closely related to the yield of RCOO (the correlation coefficients were greater than 0.9). Therefore, under optimal conditions, the yield of RCOO and principal UFAs always reached the optimal value at the same time. Based on the above results, this work realized the optimization of CO_2_-SFE green extraction process and the confirmation of principal bioactive ingredients of the extract, which laid a foundation for the green production of RCOO.

## 1. Introduction

**Figure 1:**
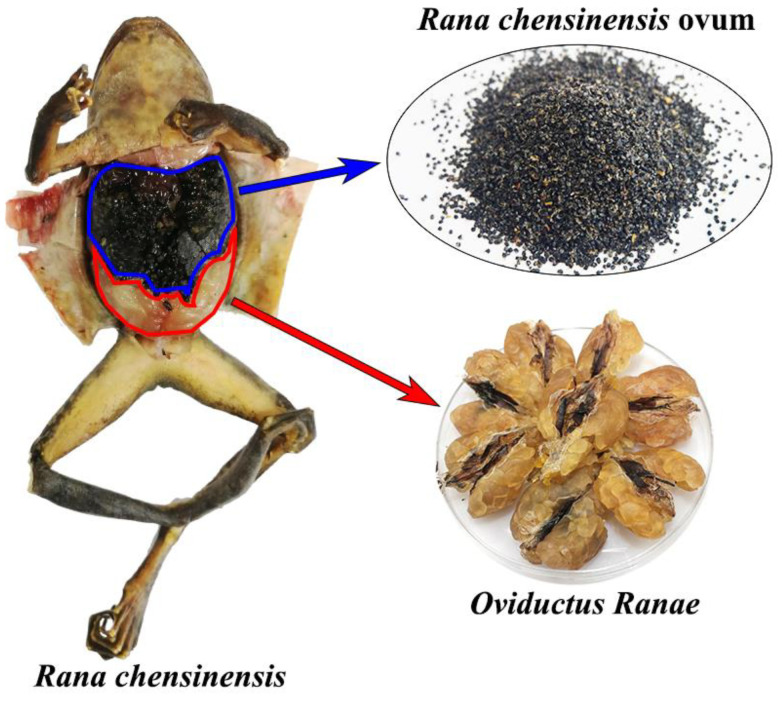
The distribution of *Rana chensinensis* ovum (RCO) and *Oviductus Ranae* in *Rana chensinensis.*

In the *Rana chensinensis* industry, *Rana chensinensis* ovum (RCO) is the residual by-product in the production process of *Oviductus Ranae* (Figure 1) [1,2,3,4,5,6]. Due to the relatively small amount of research on the by-products, RCO is usually treated as waste, causing serious wastage of *Rana chensinensis* resources. In our previous research, we found that *Rana chensinensis* ovum oil (RCOO) was rich in a variety of unsaturated fatty acids (UFAs), and its UFA content is several times higher than that of *Oviductus Ranae* [7]. Currently, UFAs are attracting much attention due to their extensive physiological activities [8,9]. For example, eicosapentaenoic acid (EPA) and docosahexaenoic acid (DHA) are closely related to the development of the vision and nervous systems [10,11]. In addition, UFAs also play an important role in the prevention and treatment of hyperlipidemia, thrombosis, and other cardiovascular and cerebrovascular diseases [12,13]. The abundant UFAs in RCOO lend it application prospects. It not only provides an emerging source of UFAs, but also effectively improves the use rate of by-products of *Rana chensinensis* resources to promote the green and sustainable development of the *Rana chensinensis* industry.

To achieve the potential value of RCOO, it is urgent to develop a green and efficient extraction technology to ensure its safe edibility and the content of the effective ingredient UFAs. The existing extraction methods of RCOO include traditional Soxhlet extraction, Bligh–Dyer extraction, etc. [14,15]. These methods are classic for the extraction of lipids [16], but they require a lot of organic solvents, which is not conducive to the large-scale production of RCOO. For example, Soxhlet extraction usually uses petroleum ether as solvent, while Bligh–Dyer extraction uses more toxic chloroform and methanol as solvents [17]. Compared with the traditional RCOO extraction methods, the newly developed CO_2_ supercritical fluid extraction (CO_2_-SFE) has significant advantages by using response surface strategy. CO_2_-SFE usually uses CO_2_ as the extraction solvent instead of organic solvents, so the RCOO extract has no residual solvent substances, thereby ensuring the 100% naturalness of the extract and avoiding the pollution of organic solvents to the environment [18]. CO_2_-SFE can be performed close to room temperature (the extraction solvent CO_2_ is in a supercritical state when the temperature is higher than 31.1 °C and the pressure is greater than 7.38 MPa) and under the envelope of CO_2_ gas, which can effectively prevent the oxidative degradation of important UFAs in RCOO [18,19]. In addition, the density of supercritical fluid is 100–1000 times that of gas, which is close to liquid, so it has similar solubility to liquid. However, its diffusion coefficient is 10–100 times that of liquid, which leads to it having a strong penetration ability into RCO, so CO_2_-SFE can obtain an efficient separation effect [20]. The surface response strategy is used to analyze the influence of various factors (for example, pressure, flow, temperature, and time) in the CO_2_-SFE process on the yield of RCOO, which can further improve the efficiency of RCOO with a relatively small number of experiments and save the development and production costs [21,22,23].

The goal of this work is to develop an innovative green and efficient CO_2_-SFE technology for RCOO, and at the same time clarify the principal UFA composition of RCOO, so as to improve the overall use rate of the by-product of *Rana chensinensis* resources. To achieve this goal, this work used a response surface methodology (RSM) based on the Box–Behnken design (BBD) to establish a response surface model for the influence of pressure, flow, temperature and time on the yields of extracts, which was used to optimize various parameters in the CO_2_-SFE process of RCOO. The biologically active ingredients in RCOO are the basis for its development and use. This work identified and quantitatively analyzed the principal UFA composition in RCOO, and at the same time studied the relationship between the yield of the principal UFAs and the yield of RCOO.

## 2. Results and Discussions

### 2.1. Adaptability Evaluation of BBD Model

The weight of RCOO is shown under different combinations of CO_2_-SFE experimental conditions in Figure S1. An amount of 300 g of RCO powder was used for each CO_2_-SFE, and the obtained RCOO showed a yellow oily liquid at room temperature. In these 27 groups of experiments, the weight of RCOO was between 7.08 and 39.17 g, and the highest yield was more than five times the lowest yield. Under different CO_2_-SFE experimental conditions, there were significant differences in the yield of RCOO, which indicated that it was necessary to explore the effects of pressure, flow, temperature, and time on the yield of RCOO.

The experimental design matrix of four factors and three levels based on BBD and the yield (%) are shown in Table 1. The response surface quadratic model was established based on the experimental data (Table 1). To explore the rationality of the model and the influence of the four independent factors (pressure, flow, temperature, and time) on the yield of RCOO in the CO_2_-SFE process, the analysis of variance (ANOVA) of the quadratic model are shown in Table 2.

The adaptability of the model was evaluated according to the results of ANOVA. The Fisher test of the model had a high F-value (F-value = 25.91) and a very low *p*-value (*p*-value < 0.0001), indicating that the model was very significant. In addition, the quality of the model can also be determined by the coefficient of determination (R^2^) [24]. Theoretically, the closer R^2^ is to 1, the better the correlation between the experimental value and the predicted values [25]. In this model, the value of R^2^ was 0.9680 (very close to 1), which indicated that there was a good agreement between the experimental and predicted yields of RCOO and the model can reasonably explain the change of yield under different experimental conditions. The adjusted coefficient of determination (Adjusted R^2^ = 0.9306) and predicted coefficient of determination (Predicted R^2^ = 0.8185) were also close to 1, indicating that the measured experimental data and predicted values were highly correlated, and that the model had fully fitted the data. In the diagnosis results of the model, the normal distribution of residuals (Figure S2A) and the comparison between predicted values and experimental values (Figure S2B) both showed good linearity, which also reflected that the model could fully express the real relationship among the selected parameters [26]. On the other hand, the results of ANOVA showed that the lack of fit was not significant (*p* > 0.05). The F-value (7.38) and *p*-value (0.1252) implied that the lack of fit was not significant relative to the pure error. Non-significant lack of fit was good, which reflected the model had good predictive ability [27,28]. Regardless of the combination of the variables in the model, the model equation could predict the yield of RCOO well. Adeq Precision is a parameter that reflects the signal-to-noise ratio, and a ratio greater than 4 is expected [29]. The ratio of 17.1612 for the model indicated that the signal was sufficient to indicate that the model could be used to navigate the design space. The above results showed that the obtained response surface model was reasonable, and that it fully reflected the true relationship between the response value (the yield of RCOO) and the four independent factors (pressure, flow, temperature, and time).

### 2.2. The Influence of Various Factors in the BBD Model

After confirming the applicability of response surface model, the influence of various factors on the yield of RCOO was investigated. According to the estimated values of the coefficients of various factors shown in Table 2, the experimental data were fitted into Equation (3) through regression analysis, and the second-order polynomial model of the influence of pressure, flow rate, temperature and time on the yield of RCOO in CO_2_-SFE process was obtained (Equation (1), in terms of coded levels).

(1) $$\begin{array}{ll} Y\;= & 11.31\;+\;2.25X_{1}\;+\;1.97X_{2}\;+\;0.007X_{3}\;+\;3.50X_{4}\;-\;1.20X_{1}X_{2}\\ & +\;1.67X_{1}X_{3}-\;0.81X_{1}X_{4}\;-\;0.38X_{2}X_{3}\;-\;1.18X_{2}X_{4}\;\\ & +\;0.38X_{3}X_{4}\;-\;1.04{X_{1}}^{2}\;-\;1.10{X_{2}}^{2}\;-\;1.20{X_{3}}^{2}\;-\;1.79{X_{4}}^{2} \end{array}$$

In this equation, Y is the response variable (the yield of RCOO). X_1_, X_2_, X_3_, and X_4_ are the coded values of the independent variables pressure, flow, temperature, and time, respectively.

In the BBD model, the significance of each model term coefficient could be determined by F-value and *p*-value (Table 2). The value of *p* < 0.05 indicated that the influence of the model term on the yield of RCOO was significant, and the larger F-values and the smaller the *p*-values, the greater the significance of the corresponding model item [30]. According to the results shown in Table 2, in the linear terms, pressure (X_1_), flow (X_2_), and time (X_4_) had extremely significant (*p* < 0.0001) effects on the yield of RCOO, while the temperature term (X_3_) was not significant (*p* = 0.9802), so its effect on the yield of RCOO was very small. Among the six interaction terms, the interaction term of pressure and flow (X_1_X_2_, *p* = 0.0218), the interaction term of pressure and temperature (X_1_X_3_, *p* = 0.0033), and the interaction term of flow and time (X_2_X_4_, *p* = 0.0236) had significant effects on the yield. Interestingly, the quadratic terms of the four independent variables (X_1_^2^, X_2_^2^, X_3_^2^, and X_4_^2^) all had a significant negative impact on the yield (*p* < 0.05). In a comprehensive analysis of each model item, although temperature had no significant linear effect on the yield of RCOO, the interaction term of X_1_X_3_ (*p* = 0.0033) and the quadratic term of temperature (X_3_^2^, *p* = 0.0105) had significant effects on the yield, indicating that temperature was also an indispensable factor. 

**Figure 2:**
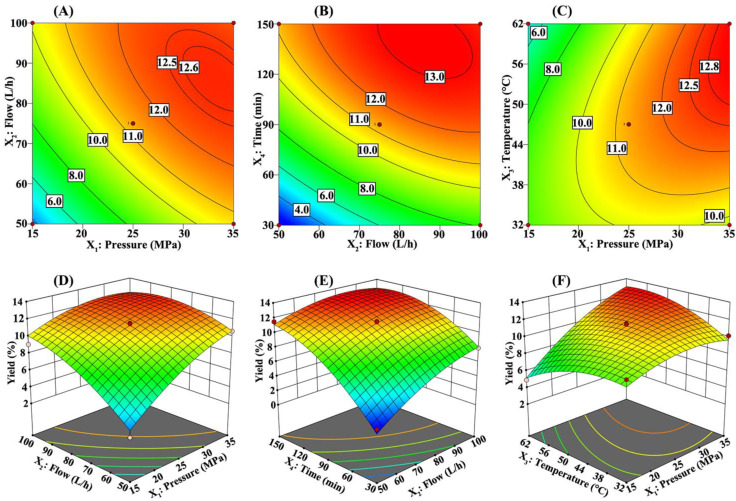
Two-dimensional (2D) contour graphs and three-dimensional (3D) surface graphs of the significant interaction items in the response surface model. (**A**,**D**) 2D contour graphs and 3D surface graphs showing the effects of pressure (X_1_) and flow (X_2_) on the yield at fixed temperature (X_3_ = 47 °C) and time (X_4_ = 90 min), respectively. (**B**,**E**) 2D contour graphs and 3D surface graphs showing the effects of flow (X_2_) and time (X_4_) on the yield at fixed pressure (X_1_ = 25 MPa) and temperature (X_3_ = 47 °C), respectively. (**C**,**F**) 2D contour graphs and 3D surface graphs showing the effects of pressure (X_1_) and temperature (X_3_) on the yield at fixed flow (X_2_ = 75 L/h) and time (X_4_ = 90 min), respectively.

In addition, two-dimensional (2D) contour graphs and three-dimensional (3D) surface graphs of the response surface model provide a visual method for directly reflecting the relationship between different variables [24]. The shape of the 2D contour graphs (an ellipse represents significant, while nearly circular represents insignificant) reflects whether the interaction between two independent variables is significant [24,31], while the 3D surface graphs can reflect the influence of two independent variables on the response value at the same time. In this work, Figure 2A, Figure 2B and Figure 2C show 2D contour graphs of the interaction term of pressure and flow (X_1_X_2_), the interaction term of flow and time (X_2_X_4_), and the interaction term of pressure and temperature (X_1_X_3_), respectively. These three 2D contour graphs all have an elliptical distribution, indicating that these interactions between the corresponding two independent variables is significant. The other three interaction terms are shown in the Supplementary Material Figure S3. Their approximately circular 2D contour graphs indicate that the interaction between the corresponding variables is negligible. The intuitive results shown in the 2D contour map are consistent with the *p*-value results shown in Table 2, which verifies the accuracy of the results in many respects.

The 3D surface graphs of the effect of pressure (X_1_) and flow (X_2_) on the yield of RCOO at a fixed temperature (X_3_ = 47 °C) and time (X_4_ = 90 min) are shown in Figure 2D. At the given flow, the increase in pressure will increase the density of the supercritical solvent CO_2_, leading to an increase in the solubility of CO_2_; thus, the yield of RCOO increases significantly with the increase in pressure. However, the effect of the negative quadratic term of pressure increased significantly at high pressure (pressure greater than 30 MPa). A possible reason is that the highly compressed CO_2_ led to the decrease in the fluid diffusion coefficient [28]. This offset effect resulted in high pressure with little effect on the yield of RCOO. At the same time, considering that extreme high-pressure conditions seriously affect the life of the instrument, it is not always recommended to use high-pressure extraction. Under the given pressure, the increase of CO_2_ flow could increase the cycle extraction times in the pipeline, thus increasing the yield of RCOO. However, when the CO_2_ flow was higher than 80 L/h, the residence time of CO_2_ was too short, so the contact time with the powder of RCO was reduced, which was not conducive to improving the yield [32]. The 3D surface graphs of the effect of flow (X_2_) and time (X_4_) on the yield at a fixed pressure (X_1_ = 25 MPa) and temperature (X_3_ = 47 °C) are shown in Figure 2E. At the specified flow, during the first 120 min of extraction time, the yield of RCOO increased significantly; when the time was longer than 120 min, the influence of time was weakened, and the yield also approached a stable state. Figure 2F shows the 3D surface graphs of the effect of pressure (X_1_) and temperature (X_3_) on the yield at a fixed flow (X_2_ = 75 L/h) and time (X_4_ = 90 min). The effect of temperature on the yield of RCOO is complex, and its effect on the yield was significantly related to the pressure. In the lower pressure range (pressure < 25 MPa), the low temperature was beneficial to improving the yield, while in the higher pressure range (pressure > 25 MPa), the effect of temperature was reversed, and the temperature increase was beneficial to improving the yield. The reverse effect of temperature on the yield of RCOO could be explained [33]. Increasing the temperature reduces the density of the CO_2_ solvent, thereby reducing its solubility. On the other hand, increasing the temperature also increasing the mass transfer and vapor pressure of solute, thus increasing its solubility in supercritical solvent. Finally, whether increasing the temperature produced a positive effect or a negative effect was dependent on whether the dominant position was the solvent density or the solute vapor pressure [21].

### 2.3. Improvement of Yield Based on the BBD Model

In the experiment of the building model, the yield of RCOO was between 2.36–13.06%. Although the range of independent variables could be customized to obtain a higher expected value of yield during the RSM optimization process, the range of experimental conditions given in this work represented an excellent compromise among the yield, the bearing range of CO_2_-SFE instrument and the production cost [34]. Therefore, in the optimization function of Design-Export software, the pressure range was set to 15–35 MPa, the flow range was set to 50–100 L/h, the temperature range was set to 32–62 °C, and the time range was set to 30–150 min. The final yield of RCOO obtained through RSM optimization was 13.61% (this yield was higher than the highest yield obtained in the modeling experiment, which was 13.06%), and the corresponding optimized production conditions were pressure 29 MPa, flow 82 L/h, temperature 50 °C, and time 132 min.

Under the optimal CO_2_-SFE conditions, 300 g of RCO powder was used as the raw material for each CO_2_-SFE and repeated three times in parallel to verify the accuracy of the model optimization results. The verification results are listed in Table S1. The actual optimal yield of RCOO was 13.29 ± 0.37%, and the average error with the predicted value was 0.38 ± 0.27%. The above results showed that the predicted value of the model is close to the actual value and the average mean error was acceptable, which confirmed that the prediction of the response surface model was accurate.

### 2.4. Identification of Principal UFAs of RCOO

**Figure 3:**
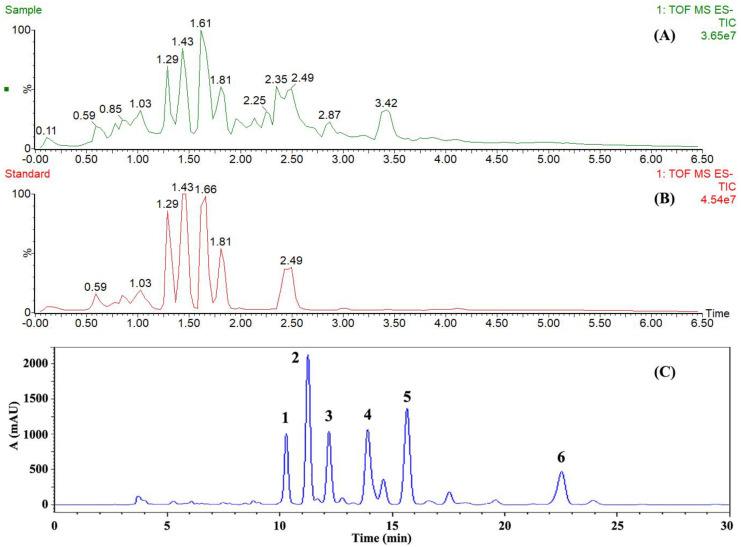
The mass spectrometry and HPLC chromatogram of *Rana chensinensis* ovum oil (RCOO). (**A**) The total ion current (TIC) chromatogram of RCOO sample. (**B**) The total ion current (TIC) chromatogram of unsaturated fatty acid (UFA) standards. (**C**) The HPLC chromatogram of RCOO sample.

After UPLC-ESI-Q-TOF-MS analysis, the total ion current (TIC) chromatogram of RCOO is shown in Figure 3A. Combined with the HPLC determination results of UFAs in the RCOO sample (Figure 3C), there were six principal peaks (peak 1–6) in the HPLC chromatogram, so the six principal UFAs corresponding to the six peaks were analyzed using mass spectrometry. According to the large amount of data provided by the comparative analysis platform and the NIST mass spectral database, the calculated m/z, molecular formula, and suggested compounds are listed in Table 3. The standards of UFAs (including EPA, ALA, DHA, ARA, LA, and OA) were made into mixed standard solutions, and mass spectrometry analysis was performed under the same conditions to verify the accuracy of the compounds in the sample (Figure 3B). In the ion peak chromatograms, the sample and the standard had the same retention time and fragment ion peaks (Figure S4), and the retention time of the sample and the standard in the HPLC chromatogram was also consistent (Figure S5). All of these indicated that the speculation result of the UFAs compounds in the sample was reasonable.

Under optimal extraction conditions (Table S1), the total content of the principal UFAs in RCOO alone reached 317.27 ± 1.54 mg/g, which was the basis of its development and application. Hamilton et al. reported that EPA and DHA were currently mainly derived from marine fisheries, which were in short supply in most parts of the world [11]. The development and use of EPA (14.42 ± 0.07 mg/g) and DHA (14.13 ± 0.05 mg/g) in RCOO not only improved the use rate of by-products, but also provided an emerging source of EPA and DHA while reducing waste. ALA and LA acid are both essential polyunsaturated fatty acids (PUFAs) for the human body [35], which may be related to reducing inflammation and reducing the risk of heart attack, and the ALA (57.70 ± 0.22 mg/g) and LA (65.45 ± 0.37 mg/g) in RCOO could be supplemented in human diet. ARA (23.19 ± 0.14 mg/g) in RCOO could be used as the direct precursor for the synthesis of prostaglandins, leukotrienes, and other bioactive substances in the human body [36,37]. OA was the most abundant UFA in olive oil (700–850 mg/g) [38,39]. Although the content of OA (142.39 ± 1.01 mg/g) in RCOO was lower than that in olive oil, it could still be used as a supplement to the conventional way of obtaining OA due to its low production costs.

### 2.5. Yield Analysis of Principal UFAs

**Figure 4:**
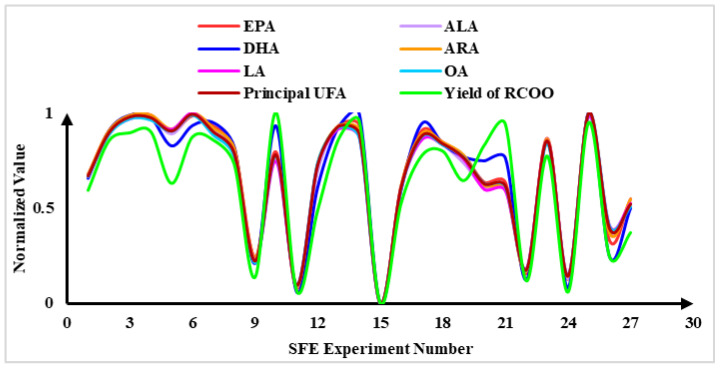
The change trend of the yield of principal unsaturated fatty acids (UFAs) and oil of *Rana chensinensis ovum* (RCOO) among different supercritical fluid extraction (CO_2_-SFE) experimental groups.

According to the standard curve of standard UFAs, the extraction yields of six principal UFAs and their total amount (mg/g, the weight of UFAs/the weight of RCO powder) were calculated, and the results are shown in Table S2. To intuitively reflect the change trend of the yield of UFAs and RCOO among different experimental groups, the corresponding yields were normalized, and the curve graph (Figure 4) was drawn to analyze the change rule. It can be found from Figure 4 that the change rule of the yields of the six principal UFAs and their total yield was consistent with the change rule of the yield of RCOO in the CO_2_-SFE extraction process. Among them, the most significant consistency was that the experimental groups 9, 11, 15, 22 and 24 all showed extremely low extraction efficiency, while experimental groups 10, 22 and 25 all showed high extraction efficiency. This may be due to their similar long-chain structure and polarity. The six principal UFAs in RCOO all belonged to long-chain UFAs, and their polarity was very small [40,41]. Therefore, according to the theory that similar dissolve mutually, when non-polar CO_2_ was used as SFE solvent, they were always extracted at the same time in the CO_2_-SFE process. In the Pearson correlation analysis (Table S3), the yield of six principal UFAs and their total yield showed a strong correlation with the yield of RCOO. All correlation coefficients were greater than 0.9, which was consistent with the change rule reflected in Figure 4. In the model validation experiment (Table S1), the yield of RCOO was the best under the optimal CO_2_-SFE conditions, and the yield of UFAs was also always higher than that of 27 groups of modeling experiments. This strong correlation indicated that the response surface model based on the yield of RCOO could also reflect the yield of the principal UFAs in RCO, which ensured the content of bioactive ingredients in RCOO. In this way, there was no need to model the yield of each UFA separately, which provided a theoretical basis for simplifying the optimization operation of RCOO in the CO_2_-SFE process.

## 3. Materials and Methods

### 3.1. Reagents and Samples

SFE solvent CO_2_ gas (purity 99.5%) was purchased from Changchun ZhongSheng Gas Co., Ltd. (Changchun, China). Acetonitrile (HPLC-grade) and methanol (HPLC-grade) were purchased from Fisher (Fisher Scientific, Fair Lawn, NJ, USA). The standards of EPA, DHA, and ARA were purchased from TanMo Quality Testing Technology Co., Ltd. (Beijing, China). The standards of α-linolenic acid (ALA), linoleic acid (LA), and oleic acid (OA) were purchased from ANPEL Laboratory Technologies (Shanghai) Inc. (Shanghai, China). The sample of RCO was purchased from the main production area of the Changbai Mountain areas (Huadian, China), then it was stored in a refrigerator at −20 °C after being natural air dried.

### 3.2. Sample Preparation

The RCO was taken out of the refrigerator at −20 °C, and then it was dried in a 45 °C blast drying oven for 24 h to remove the residual moisture. The dried RCO was crushed by the hammer crusher, and the particle size of powder was controlled at between 0.2–1 mm through screen filtration.

### 3.3. CO_2_-SFE Procedure

**Figure 5:**
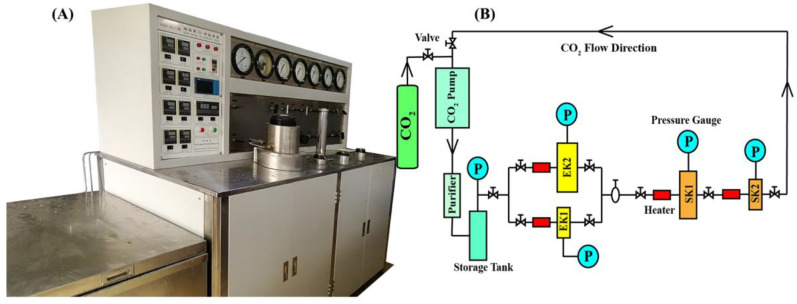
The CO_2_ supercritical fluid extraction (CO_2_-SFE) equipment. (**A**) The HA221-40-11 CO_2_-SFE device used in this work. (**B**) The diagrammatic sketch of working principle of CO_2_-SFE equipment. EK1 and EK2 are two parallel extraction kettles. SK1 and SK2 are two separation kettles in series.

The HA221-40-11 CO_2_ supercritical fluid extraction equipment purchased from Nantong Huaan Supercritical Extraction Co., Ltd. (Nantong, Jiangsu, China) was used to extract RCOO (Figure 5A). The equipment is equipped with a refrigeration device (cooling cycle of CO_2_), temperature control and display system, pressure control and display system, purifier, mixer, heat exchanger, storage tank, CO_2_ pump, two parallel extraction kettles (the specification of extraction kettle I is 10 L/40 MPa, and the specification of extraction kettle II is 1 L/50 MPa), two separation kettles in series (the specification of separation kettle I is 4 L/30 MPa, and the specification of separation kettle II is 2 L/30 MPa), and multiple gas control valves (Figure 5B).

In this work, each time, 300 g of RCO powder was loaded into the cylindrical stainless-steel extraction tank matched with extraction kettle II. After the stainless-steel extraction tank was covered, it was put into extraction kettle II with a tool. Finally, the cabin doors of the extraction kettle and the separation kettle were closed, and the preparation work before extraction was completed. CO_2_ (purity 99.5%) was used as the extraction solvent, and the CO_2_-SFE conditions were as follows: the pressure of the extraction kettle was set to 15–35 MPa, the flow was set to 50–100 L/h, the temperature of the extraction kettle was set to 32–62 °C, and the extraction time was 30–150 min. The temperatures of separation kettle I and separation kettle II were both increased by 5 °C on the basis of the extraction kettle, and the pressure was maintained at 6 MPa. Finally, the CO_2_-SFE products were collected from the separation kettle using a 100 mL conical flask, and then they were weighed and stored in a refrigerator at −20 °C until UPLC-ESI-Q-TOF-MS component identification and HPLC analysis.

### 3.4. Experimental Design of RSM Based on BBD

In the CO_2_-SFE process, pressure, flow, temperature, and time are all important factors affecting the extraction yield [42]. Therefore, RSM based on BBD was used to study the effects of these four independent factors on the yield of RCOO. The BBD model used in this work was four-factor and three-level (Table 4), in which the center point was repeated three times, and a total of 27 groups of experiments were performed. The yield of RCOO was used as the response variable of the experiments, which was calculated according to Equation (2).

(2) $$\mathrm{Yield}\;(\%)\;=\;\frac{m_{\mathrm{oil}}}{m_{\mathrm{sample}}}\;\times \;100$$

In this equation, m_oil_ and m_sample_ are the weight of RCOO (g) and the weight of RCO (g), respectively. The entire RSM experiment was carried out according to the experimental sequence designed by Design-Export (version 11, StatEase, Inc., Minneapolis, MN, USA). After data collection was completed, the experimental data were fitted into the second-order polynomial model (Equation (3)) through regression analysis, and the regression coefficient of the model was obtained.

(3) $${Y\;=\;\beta }_{0}\;+\;\sum_{i\;=\;1}^{n}\beta_{i}X_{i}\;+\;\sum_{i\;=\;1}^{n}\beta_{\mathrm{ii}}X_{i}^{2}\;+\;\sum_{i\;=\;1}^{n-1}\sum_{j\;=\;i+1}^{n}\beta_{\mathrm{ij}}X_{i}X_{j}$$

where Y is the response variable, X_i_ and X_j_ are different independent variables, and n is the number of independent variables (n = 4 in this work). β_0_ is a constant coefficient, β_i_ is the coefficient of the linear term, β_ii_ is the coefficient of the quadratic term, and β_ij_ is the coefficient of the interaction term. After building the model, the analysis of the model, response surface graph and ANOVA statistics were performed with Design-Expert at a confidence interval of 95% (*p* < 0.05) [43].

### 3.5. UPLC-ESI-Q-TOF-MS Component Analysis

UPLC-ESI-Q-TOF-MS analysis was performed on a Waters Xevo G2-XS QTOF mass spectrometer (Milford, MA, USA) equipped with a UPLC system and an electrospray ionization source (ESI). The column was Waters ACQUITY UPLC BEH C_18_ (100 × 2.1 mm, 1.7 μm) (Milford, MA, USA), and the temperature of the column was set at 30 °C. The mobile phases consisted of eluent A (0.1% formic acid acetonitrile solution, v/v) and eluent B (0.1% formic acid aqueous solution, v/v), and the flow rate was 0.4 mL/min. The gradient elution conditions were as follows: 0–2 min, linear gradient 86–93% A; 93% A in 2–8 min. The mass spectrometry analysis was performed using an ESI, and the accurate molecular mass was corrected by leucine enkephalin solution. The negative ion mass spectrometry scanning mode was used for detection. The scanning m/z range was 100–1200 Da, the scanning time was 0.2 s, the sprayer flow rate was 800 L/h, and the cone voltage was set to 40 V. The sample of RCOO was dissolved in methanol (through 0.22 μm microporous membrane) and analyzed by mass spectrometry. The mass spectrometry data were collected and recorded by Masslynx workstation (version 4.1, waters, Manchester, UK).

### 3.6. HPLC Analysis

An Agilent 1260 series liquid chromatograph (Palo Alto, CA, USA) was used to measure the content of UFAs in RCOO. In this work, the chromatographic column used was Agilent TC-C_18_ column (250 × 4.6 mm, 5 μm), and the column temperature was set to 30 °C. The mobile phase A was HPLC-grade acetonitrile, and the mobile phase B was 1% phosphoric acid solution (v/v). In the gradient elution program, the mobile phase change conditions were 0–10 min, linear gradient 86–93% A; 93% A in 10– 20 min; 20–30 min, linear gradient 93–100% A. The flow rate change conditions were 0–14 min, linear gradient 1.0–0.5 mL/min and 0.5 mL/min in 14–30 min. The detection wavelength was 203 nm. The sample solution and the UFAs standard solution (through 0.22 μm microporous membrane) were measured under the same HPLC conditions. Agilent Chemstation software (Palo Alto, CA, USA) was used to record and process data.

## 4. Conclusions

This work successfully established a green and efficient CO_2_-SFE technology for RCOO, which provides a solution for the green production of RCOO and lays a foundation for improving the comprehensive use rate of *Rana chensinensis* resources. The response surface strategy was used to guide the establishment of the Box–Behnken Design (BBD) model, and the fully fitting model enabled RCOO to obtain an optimal yield under relatively mild conditions, which saved the development and production costs of RCOO. The six kinds of principal UFA (including EPA, ALA, DHA, ARA, LA, and OA) in RCOO identified by UPLC-ESI-Q-TOF-MS made it a promising emerging source of UFAs. Simultaneously, these UFAs of growing concern have a wide range of physiological activities, which improves the value of the development and application of RCOO. In addition, the CO_2_-SFE technology developed in this work was not only limited to the extraction RCOO, but could also expand the application scope of CO_2_-SFE technology, providing a theoretical basis for the development and use of similar products.

## Figures and Tables

**Table 1:** The experimental conditions of CO_2_ supercritical fluid extraction (CO_2_-SFE) and yield of *Rana chensinensis* ovum oil (RCOO) based on the Box–Behnken Design (BBD).

No.	X_1_: Pressure (MPa)	X_2_: Flow (L/h)	X_3_: Temperature (°C)	X_4_: Time (min)	Actual Yield (%)	Predicted Yield (%)	Residual (%)
SFE1	35	75	47	30	8.69	8.05	0.64
SFE2	25	75	47	90	11.48	11.31	0.17
SFE3	35	100	47	90	11.92	12.19	−0.27
SFE4	25	100	32	90	11.99	11.36	0.63
SFE5	15	100	47	90	9.08	10.09	−1.01
SFE6	25	100	62	90	11.69	10.61	1.08
SFE7	25	50	47	150	11.51	11.12	0.39
SFE8	35	75	32	90	10.16	9.65	0.51
SFE9	25	75	32	30	3.81	5.20	−1.39
SFE10	35	75	47	150	13.06	13.42	−0.36
SFE11	15	75	47	30	3.03	1.92	1.11
SFE12	25	50	62	90	7.55	7.42	0.13
SFE13	25	75	47	90	11.55	11.31	0.24
SFE14	25	75	62	150	12.56	12.20	0.36
SFE15	25	50	47	30	2.36	1.77	0.59
SFE16	25	100	47	30	7.96	8.07	−0.11
SFE17	15	75	47	150	10.65	10.53	0.12
SFE18	25	75	47	90	10.89	11.31	−0.42
SFE19	15	75	32	90	9.24	8.48	0.76
SFE20	25	75	32	150	11.25	11.44	−0.19
SFE21	25	100	47	150	12.39	12.71	−0.32
SFE22	25	75	62	30	3.61	4.46	−0.85
SFE23	35	50	47	90	10.62	10.65	−0.03
SFE24	15	50	47	90	2.98	3.74	−0.76
SFE25	35	75	62	90	12.51	13.00	−0.49
SFE26	15	75	62	90	4.93	5.16	−0.23
SFE27	25	50	32	90	6.32	6.65	−0.33

X_1_, X_2_, X_3_, and X_4_ are the codes for pressure, flow, temperature, and time in the BBD model, respectively.

**Table 2:** The results of analysis of variance (ANOVA) of the response surface quadratic model.

Source	Coefficient Estimate	Sum of Squares	df	Mean Square	F-value	*p*-value	Significance
Model	N/A	301.02	14	21.50	25.91	<0.0001	**
Intercept	11.31	N/A	N/A	N/A	N/A	N/A	N/A
X_1_: Pressure	2.25	60.98	1	60.98	73.49	<0.0001	**
X_2_: Flow	1.97	46.77	1	46.77	56.36	<0.0001	**
X_3_: Temperature	0.007	0.0005	1	0.0005	0.0006	0.9802	not significant
X_4_: Time	3.50	146.72	1	146.72	176.82	<0.0001	**
X_1_ X_2_	−1.20	5.76	1	5.76	6.94	0.0218	*
X_1_X_3_	1.67	11.09	1	11.09	13.36	0.0033	**
X_1_X_4_	−0.81	2.64	1	2.64	3.18	0.0997	not significant
X_2_X_3_	−0.38	0.59	1	0.59	0.71	0.4174	not significant
X_2_X_4_	−1.18	5.57	1	5.57	6.71	0.0236	*
X_3_X_4_	0.38	0.57	1	0.57	0.69	0.4234	not significant
X_1_^2^	−1.04	5.76	1	5.76	6.94	0.0218	*
X_2_^2^	−1.10	6.47	1	6.47	7.80	0.0163	*
X_3_^2^	−1.20	7.62	1	7.62	9.19	0.0105	*
X_4_^2^	−1.79	17.05	1	17.05	20.55	0.0007	**
Residual	N/A	9.96	12	0.83	N/A	N/A	N/A
Lack of Fit	N/A	9.69	10	0.97	7.38	0.1252	not significant
Pure Error	N/A	0.26	2	0.13	N/A	N/A	N/A
Cor Total	N/A	310.98	26	N/A	N/A	N/A	N/A
R^2^ = 0.9680		Adjusted R^2^ = 0.9306		Predicted R^2^ = 0.8185		Adeq Precision = 17.1612	

X_1_, X_2_, X_3_, and X_4_ are the codes of pressure, flow, temperature, and time in the BBD model, respectively. df: degree of freedom. N/A: Not applicable. * *p* < 0.05, ** *p* < 0.01.

**Table 3:** The unsaturated fatty acid (UFA) compounds corresponding to the six principal peaks in the HPLC chromatogram of *Rana chensinensis* ovum oil (RCOO).

Peak	RT in MS ^a^ (min)	[M − H]^−^	Mass Error (mDa)	Molecular Formula	Proposed Compound	RT in HPLC ^b^ (min)
1	1.287	301.1901	2.0	C_20_H_30_O_2_	EPA	10.29
2	1.400	277.1917	2.3	C_18_H_30_O_2_	ALA	11.26
3	1.467	327.2035	1.9	C_22_H_32_O_2_	DHA	12.19
4	1.614	303.2077	2.5	C_20_H_32_O_2_	ARA	13.91
5	1.806	279.2090	2.8	C_18_H_32_O_2_	LA	15.66
6	2.427	281.2235	2.1	C_18_H_34_O_2_	OA	22.53

^a^ The retention time in the total ion current (TIC) chromatogram. ^b^ The retention time in the HPLC chromatogram. EPA: eicosapentaenoic acid; ALA: α-linolenic acid; DHA: docosahexaenoic acid; ARA: arachidonic acid; LA: linoleic acid; OA: oleic acid.

**Table 4:** The factors and factor level setting of the Box–Behnken design (BBD).

Coding Level	Pressure (X_1_, MPa)	Flow (X_2,_ L/h)	Temperature (X_3_, °C)	Time (X_4_, min)
−1	15	50	32	30
0	25	75	47	90
+1	35	100	62	150

## Supplementary Materials

The following are available online, Figure S1: The weight of *Rana chensinensis* ovum oil (RCOO) produced by CO_2_ supercritical fluid extraction (CO_2_-SFE) based on Box–Behnken design (BBD), Figure S2: The diagnostic results of response surface model. (A) The normal distribution of residuals. (B) The comparison of actual and predicted values of the yield of *Rana chensinensis* ovum oil (RCOO), Figure S3: Two-dimensional (2D) contour graphs and three-dimensional (3D) surface graphs of the non-significant interaction items in the response surface model. (A, D) 2D contour graphs and 3D surface graphs showing the effects of pressure (X_1_) and time (X_4_) on the yield at fixed flow (X_2_ = 75 L/h) and temperature (X_3_ = 47 °C), respectively. (B, E) 2D contour graphs and 3D surface graphs showing the effects of flow (X_2_) and temperature (X_3_) on the yield at fixed pressure (X_1_ = 25 MPa) and time (X_4_ = 90 min), respectively. (C,F) 2D contour graphs and 3D surface graphs showing the effects of temperature (X_3_) and time (X_4_) on the yield at fixed pressure (X_1_ = 25 MPa) and flow (X_2_ = 75 L/h), respectively, Figure S4: The ion peak chromatograms of the six-principal unsaturated fatty acids (UFAs) in *Rana chensinensis* ovum oil (RCOO) sample and their standard, EPA: eicosapentaenoic acid; ALA: α-linolenic acid; DHA: docosahexaenoic acid; ARA: arachidonic acid; LA: linoleic acid; OA: oleic acid, Figure S5: The HPLC chromatogram of *Rana chensinensis* ovum oil (RCOO) sample and six kinds of unsaturated fatty acid (UFAs) mixed standards. EPA: eicosapentaenoic acid; ALA: α-linolenic acid; DHA: docosahexaenoic acid; ARA: arachidonic acid; LA: linoleic acid; OA: oleic acid, Table S1: The results of model validation, the content of principal unsaturated fatty acids (UFAs) in *Rana chensinensis* ovum oil (RCOO) and the yield of principal UFAs under the optimal conditions, Table S2: The extraction yield of six principal unsaturated fatty acids (UFAs) and their total amount, Table S3: The correlation coefficient of Pearson correlation analysis.
