# Supplementary material for: Rana chensinensis Ovum Oil Based on CO2 Supercritical Fluid Extraction: Response Surface Methodology Optimization and Unsaturated Fatty Acid Ingredient Analysis
Source: Molecules. 2020 Sep 11;25(18):4170. doi: 10.3390/molecules25184170 (PMC7570602; doi:10.3390/molecules25184170)
Supplement: Supplementary file 1 [file molecules-25-04170-s001.docx]

***Rana Chensinensis* Ovum Oil Based on CO_2_ Supercritical Fluid Extraction: Response Surface Methodology Optimization and Unsaturated Fatty Acid Ingredient Analysis**

**Yuanshuai Gan ^1^, Dongliang Xu ^1^, Jianqiu Zhang ^1^, Zhongyao Wang ^1^, Shihan Wang ^2^, Hongye Guo ^1^, Kexin Zhang ^1^, Yajing Li ^1^ and Yongsheng Wang ^1,^***

^1^ School of Pharmaceutical Sciences, Jilin University, Changchun, Jilin 130021, China; ganys18@mails.jlu.edu.cn (Y.G.); xudl19@mails.jlu.edu.cn (D.X.); jqzhang19@mails.jlu.edu.cn (J.Z.); zhongyao18@mails.jlu.edu.cn (Z.W.); guohy18@mails.jlu.edu.cn (H.G.); zhangkx2818@mails.jlu.edu.cn (K.Z.); liyj2818@mails.jlu.edu.cn (Y.L.); wys@jlu.edu.cn (Y.W.)

^2^ College of Chinese Medicine Materials, Jilin Agricultural University, Changchun, Jilin 130118, China; wsh8805@163.com

***** Correspondence: wys@jlu.edu.cn or mikewangwys@outlook.com; Tel.: +86-139-4416-5683


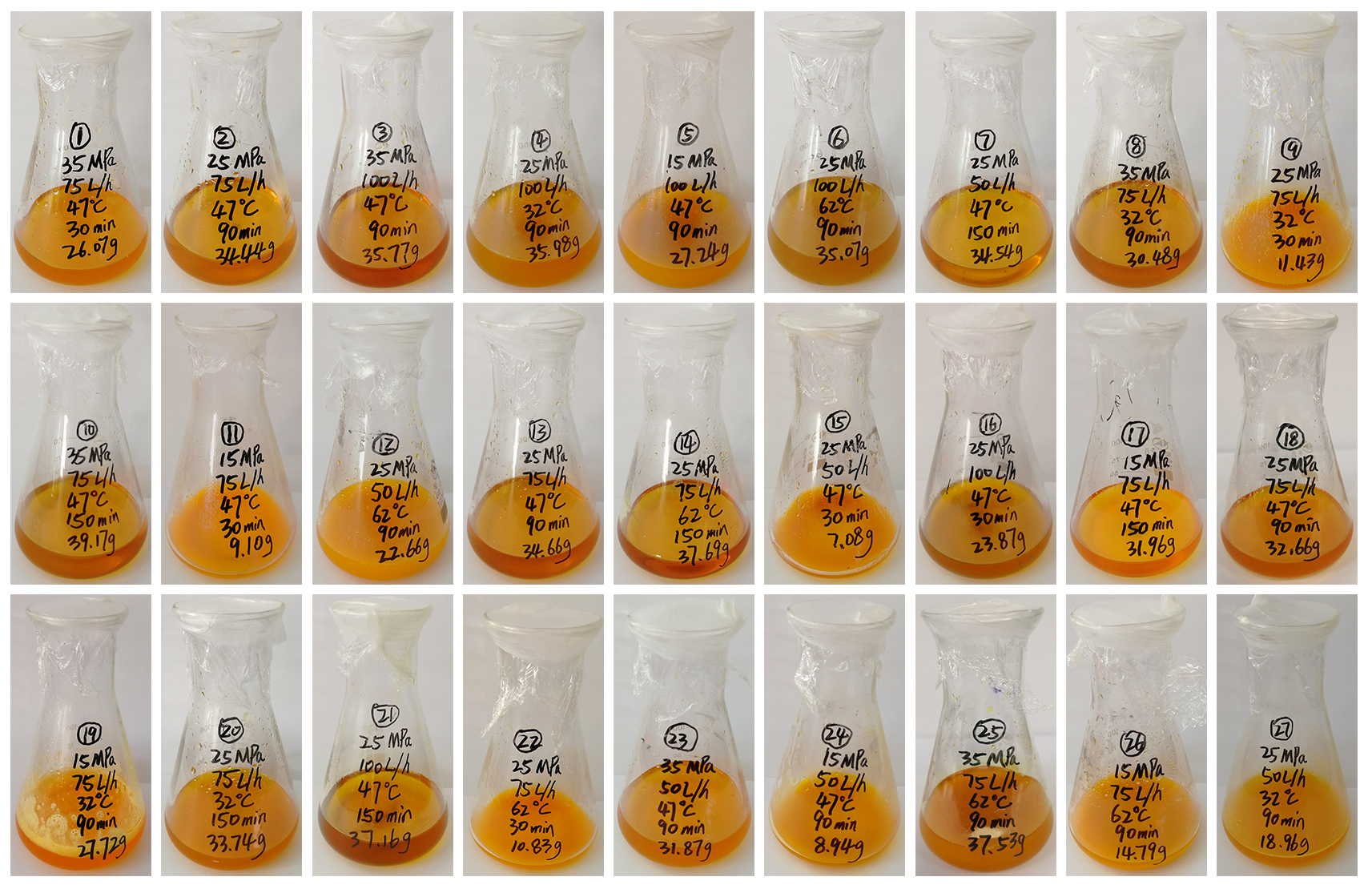


**Figure S1.** The weight of *Rana chensinensis* ovum oil (RCOO) produced by CO_2_ supercritical fluid extraction (CO_2_-SFE) based on Box-Behnken design (BBD)


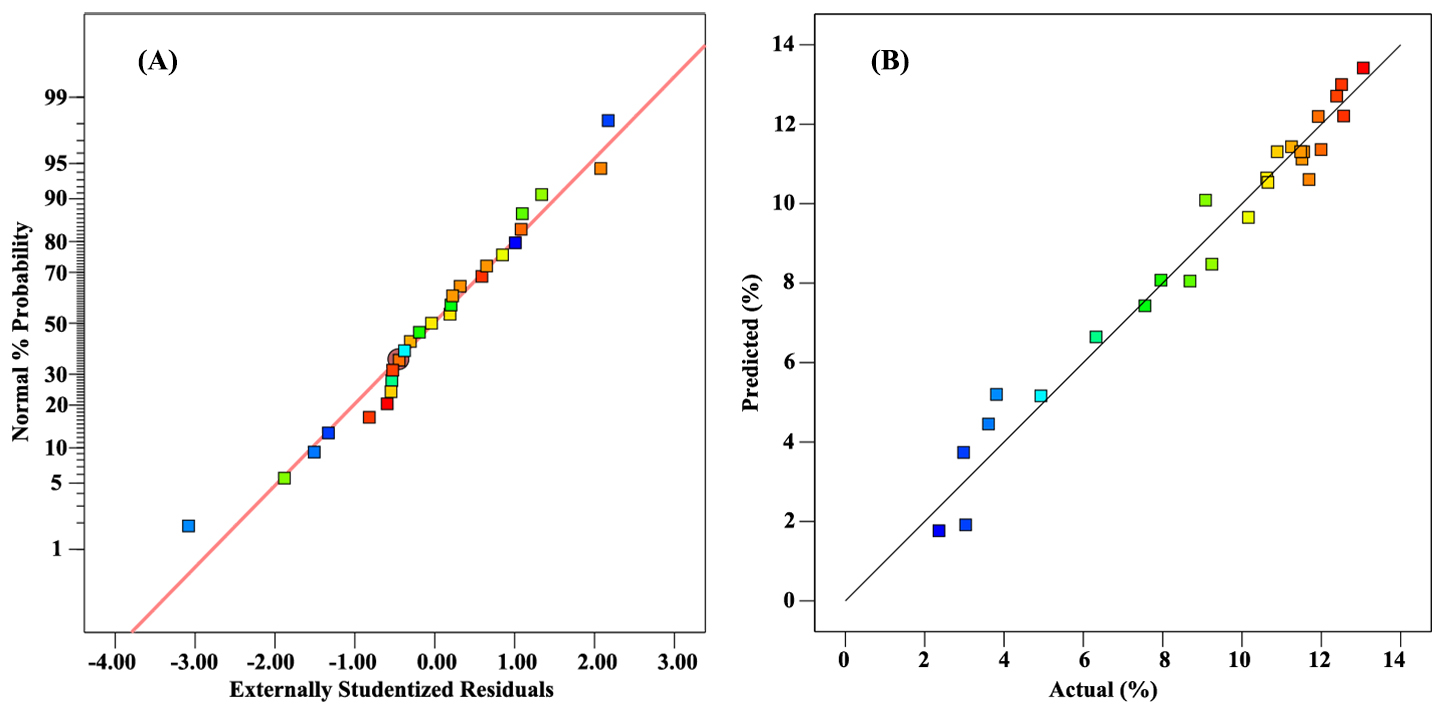


**Figure S2.** The diagnostic results of response surface model. **(A)** The normal distribution of residuals. **(B)** The comparison of actual and predicted values of the yield of *Rana chensinensis* ovum oil (RCOO).


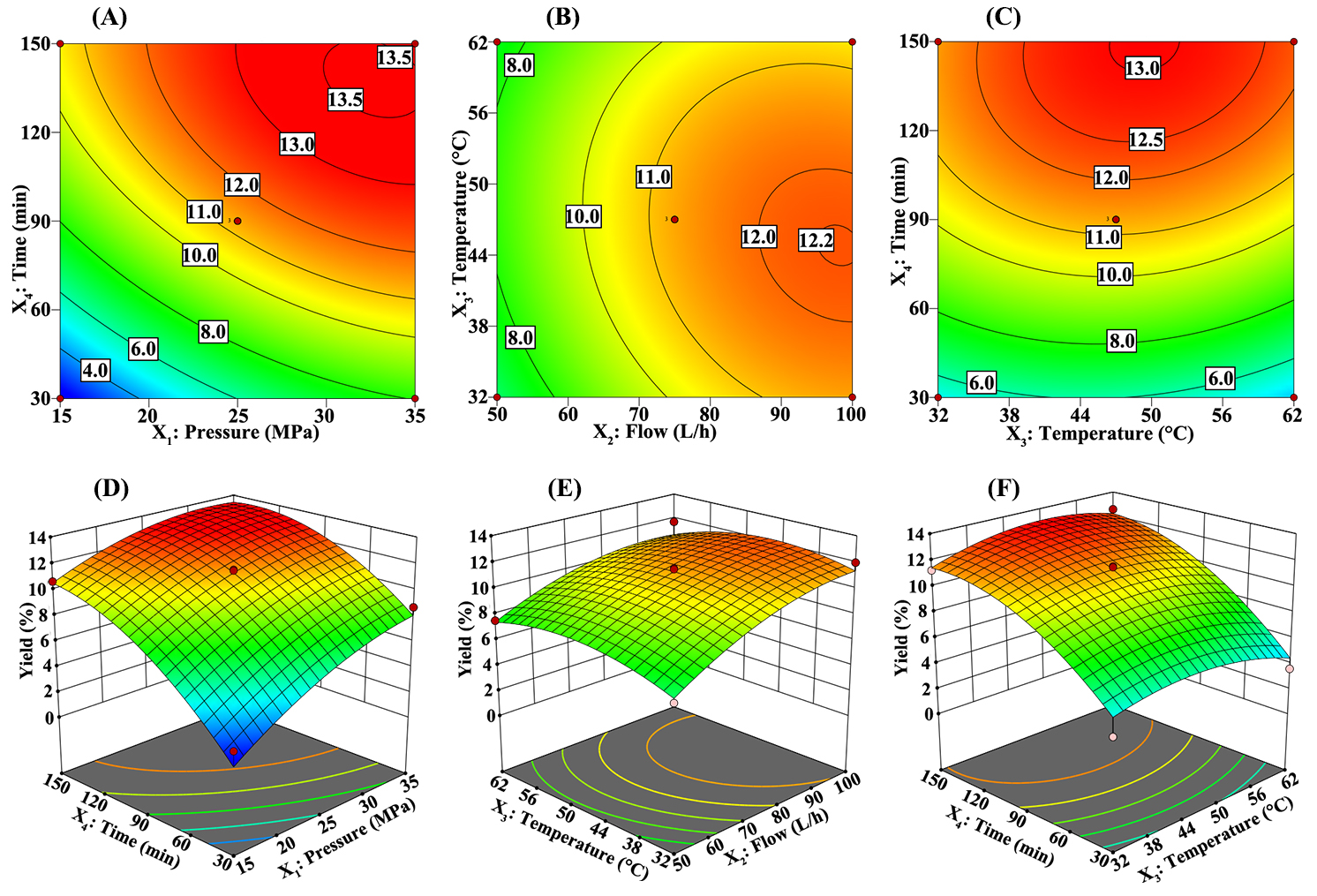


**Figure S3.** Two-dimensional (2D) contour graphs and three-dimensional (3D) surface graphs of the non-significant interaction items in the response surface model. (**A, D**) 2D contour graphs and 3D surface graphs showing the effects of pressure (X_1_) and time (X_4_) on the yield at fixed flow (X_2_ = 75 L/h) and temperature (X_3_ = 47 °C), respectively. (**B, E**) 2D contour graphs and 3D surface graphs showing the effects of flow (X_2_) and temperature (X_3_) on the yield at fixed pressure (X_1_ = 25 MPa) and time (X_4_ = 90 min), respectively. (**C, F**) 2D contour graphs and 3D surface graphs showing the effects of temperature (X_3_) and time (X_4_) on the yield at fixed pressure (X_1_ = 25 MPa) and flow (X_2_ = 75 L/h), respectively.


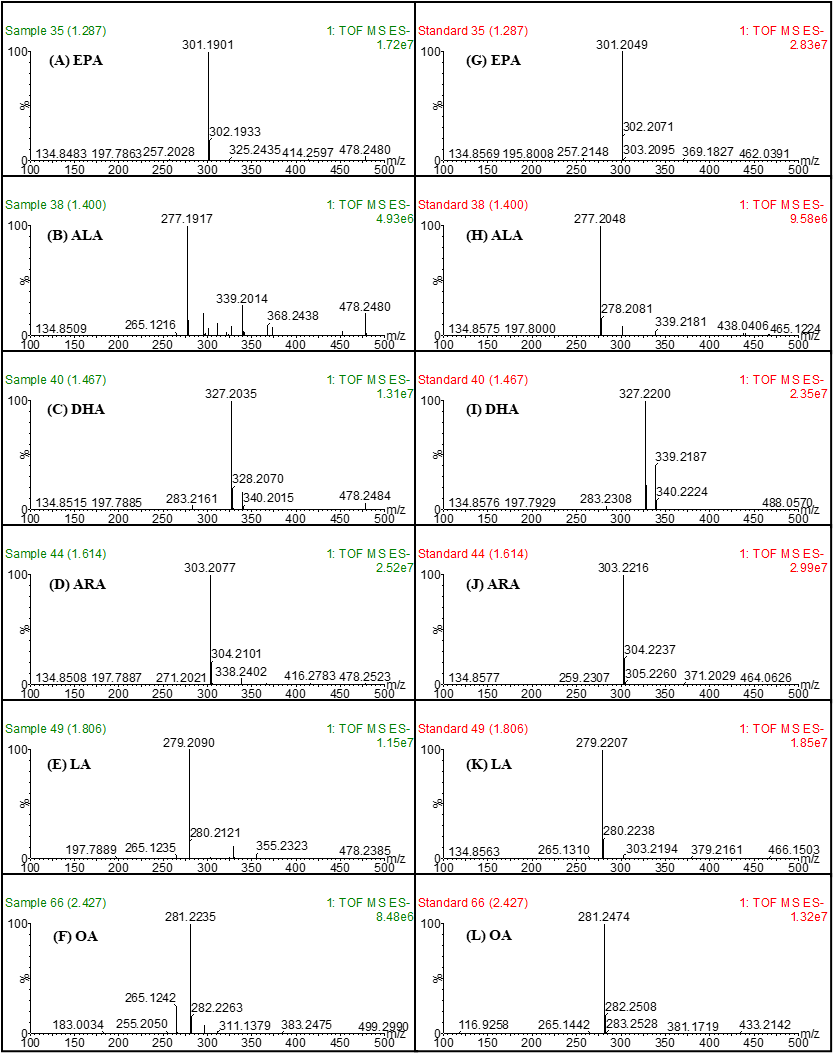


**Figure S4.** The ion peak chromatograms of the six-principal unsaturated fatty acids (UFAs) in *Rana chensinensis* ovum oil (RCOO) sample and their standard. EPA: eicosapentaenoic acid; ALA: α-linolenic acid; DHA: docosahexaenoic acid; ARA: arachidonic acid; LA: linoleic acid; OA: oleic acid.


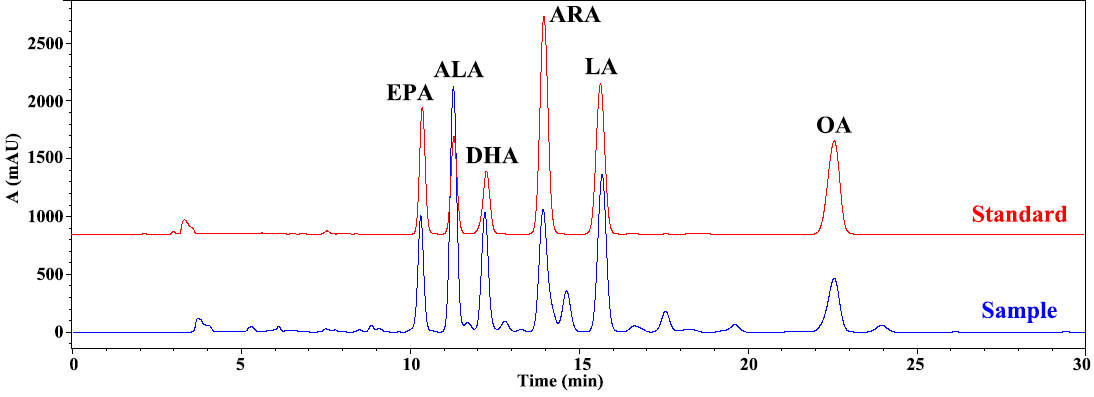


**Figure S5.** The HPLC chromatogram of *Rana chensinensis* ovum oil (RCOO) sample and six kinds of unsaturated fatty acids (UFAs) mixed standards. EPA: eicosapentaenoic acid; ALA: α-linolenic acid; DHA: docosahexaenoic acid; ARA: arachidonic acid; LA: linoleic acid; OA: oleic acid.

**Table S1.** The results of model validation, the content of principal unsaturated fatty acids (UFAs) in *Rana chensinensis* ovum oil (RCOO) and the yield of principal UFAs under the optimal conditions

|  | SFE-V1 | SFE-V2 | SFE-V3 | Mean ± SD ^c^ |
| --- | --- | --- | --- | --- |
| Weight of RCOO (g) | 39.09 | 41.11 | 39.51 | 39.9 ± 1.07 |
| Predicted Yield of RCOO (%) | 13.61 | | | 13.61 |
| Actual Yield of RCOO (%) | 13.00 | 13.70 | 13.17 | 13.29 ± 0.37 |
| Residual (%) | 0.61 | 0.09 | 0.44 | 0.38 ± 0.27 |
| **Yield ^a^** | | | | |
| Yield of EPA (%) | 0.19 | 0.20 | 0.19 | 0.19 ± 0.004 |
| Yield of ALA (%) | 0.75 | 0.79 | 0.76 | 0.77 ± 0.02 |
| Yield of DHA (%) | 0.18 | 0.19 | 0.19 | 0.19 ± 0.004 |
| Yield of ARA (%) | 0.30 | 0.32 | 0.30 | 0.31 ± 0.01 |
| Yield of LA (%) | 0.86 | 0.89 | 0.86 | 0.87 ± 0.02 |
| Yield of OA (%) | 1.87 | 1.95 | 1.86 | 1.89 ± 0.05 |
| Yield of Principal UFAs (%) | 4.16 | 4.34 | 4.17 | 4.22 ± 0.10 |
| **Content ^b^** | | | | |
| Content of EPA in RCOO (mg/g) | 14.50 | 14.37 | 14.38 | 14.42 ± 0.07 |
| Content of ALA in RCOO (mg/g) | 57.64 | 57.52 | 57.95 | 57.70 ± 0.22 |
| Content of DHA in RCOO (mg/g) | 14.18 | 14.08 | 14.12 | 14.13 ± 0.05 |
| Content of ARA in RCOO (mg/g) | 23.35 | 23.14 | 23.09 | 23.19 ± 0.14 |
| Content of LA in RCOO (mg/g) | 65.87 | 65.26 | 65.21 | 65.45 ± 0.37 |
| Content of OA in RCOO (mg/g) | 143.51 | 142.12 | 141.54 | 142.39 ± 1.01 |
| Content of Principal UFAs in RCOO (mg/g) | 319.05 | 316.49 | 316.28 | 317.27 ± 1.54 |

^a^ The yield of UFAs (the weight of UFAs (g) / the weight of *Rana chensinensis* ovum (RCO) powder (g) * 100, %). ^b^ The content of UFAs in RCOO (the weight of UFAs / the weight of RCOO, mg/g). ^c^ The values were expressed as mean ± SD (standard deviation). EPA: eicosapentaenoic acid; ALA: α-linolenic acid; DHA: docosahexaenoic acid; ARA: arachidonic acid; LA: linoleic acid; OA: oleic acid.

**Table S2.** The extraction yield of six principal unsaturated fatty acids (UFAs) and their total amount

| **NO.** | **EPA (%)** | **ALA (%)** | **DHA (%)** | **ARA (%)** | **LA (%)** | **OA (%)** | **Total of UFAs (%)** |
| --- | --- | --- | --- | --- | --- | --- | --- |
| SFE1 | 0.123 ± 0.004 | 0.520 ± 0.026 | 0.115 ± 0.003 | 0.205 ± 0.007 | 0.614 ± 0.032 | 1.470 ± 0.015 | 3.047 ± 0.059 |
| SFE2 | 0.153 ± 0.005 | 0.651 ± 0.057 | 0.148 ± 0.004 | 0.255 ± 0.020 | 0.758 ± 0.050 | 1.811 ± 0.125 | 3.775 ± 0.136 |
| SFE3 | 0.163 ± 0.011 | 0.697 ± 0.048 | 0.158 ± 0.003 | 0.272 ± 0.024 | 0.809 ± 0.053 | 1.941 ± 0.103 | 4.039 ± 0.047 |
| SFE4 | 0.163 ± 0.006 | 0.696 ± 0.030 | 0.157 ± 0.005 | 0.272 ± 0.010 | 0.809 ± 0.049 | 1.930 ± 0.033 | 4.027 ± 0.008 |
| SFE5 | 0.152 ± 0.007 | 0.645 ± 0.026 | 0.137 ± 0.007 | 0.256 ± 0.014 | 0.773 ± 0.047 | 1.839 ± 0.032 | 3.802 ± 0.088 |
| SFE6 | 0.163 ± 0.004 | 0.700 ± 0.019 | 0.151 ± 0.005 | 0.274 ± 0.003 | 0.828 ± 0.065 | 1.969 ± 0.086 | 4.086 ± 0.114 |
| SFE7 | 0.156 ± 0.006 | 0.653 ± 0.017 | 0.153 ± 0.004 | 0.258 ± 0.014 | 0.762 ± 0.015 | 1.807 ± 0.063 | 3.789 ± 0.074 |
| SFE8 | 0.141 ± 0.007 | 0.594 ± 0.006 | 0.137 ± 0.009 | 0.235 ± 0.008 | 0.692 ± 0.018 | 1.633 ± 0.091 | 3.431 ± 0.062 |
| SFE9 | 0.067 ± 0.003 | 0.276 ± 0.007 | 0.057 ± 0.002 | 0.112 ± 0.003 | 0.331 ± 0.014 | 0.770 ± 0.013 | 1.613 ± 0.030 |
| SFE10 | 0.138 ± 0.003 | 0.582 ± 0.021 | 0.151 ± 0.007 | 0.226 ± 0.013 | 0.666 ± 0.037 | 1.638 ± 0.057 | 3.401 ± 0.102 |
| SFE11 | 0.046 ± 0.001 | 0.207 ± 0.011 | 0.038 ± 0.002 | 0.080 ± 0.004 | 0.249 ± 0.013 | 0.588 ± 0.006 | 1.207 ± 0.015 |
| SFE12 | 0.127 ± 0.004 | 0.538 ± 0.014 | 0.109 ± 0.005 | 0.218 ± 0.013 | 0.656 ± 0.020 | 1.580 ± 0.042 | 3.228 ± 0.040 |
| SFE13 | 0.156 ± 0.006 | 0.669 ± 0.007 | 0.150 ± 0.003 | 0.260 ± 0.012 | 0.775 ± 0.020 | 1.872 ± 0.019 | 3.882 ± 0.017 |
| SFE14 | 0.156 ± 0.002 | 0.648 ± 0.028 | 0.159 ± 0.006 | 0.254 ± 0.017 | 0.746 ± 0.034 | 1.788 ± 0.036 | 3.751 ± 0.030 |
| SFE15 | 0.035 ± 0.001 | 0.158 ± 0.007 | 0.030 ± 0.001 | 0.060 ± 0.003 | 0.187 ± 0.008 | 0.440 ± 0.013 | 0.911 ± 0.006 |
| SFE16 | 0.115 ± 0.006 | 0.492 ± 0.010 | 0.107 ± 0.003 | 0.193 ± 0.015 | 0.576 ± 0.036 | 1.374 ± 0.060 | 2.857 ± 0.068 |
| SEF17 | 0.153 ± 0.004 | 0.632 ± 0.033 | 0.153 ± 0.003 | 0.252 ± 0.007 | 0.739 ± 0.045 | 1.796 ± 0.072 | 3.723 ± 0.071 |
| SFE18 | 0.145 ± 0.001 | 0.612 ± 0.012 | 0.139 ± 0.009 | 0.242 ± 0.009 | 0.722 ± 0.025 | 1.727 ± 0.060 | 3.587 ± 0.085 |
| SFE19 | 0.136 ± 0.005 | 0.561 ± 0.020 | 0.130 ± 0.003 | 0.227 ± 0.006 | 0.673 ± 0.042 | 1.626 ± 0.033 | 3.353 ± 0.027 |
| SFE20 | 0.118 ± 0.005 | 0.490 ± 0.018 | 0.127 ± 0.004 | 0.193 ± 0.003 | 0.570 ± 0.021 | 1.412 ± 0.049 | 2.910 ± 0.057 |
| SFE21 | 0.117 ± 0.005 | 0.489 ± 0.010 | 0.129 ± 0.010 | 0.189 ± 0.010 | 0.566 ± 0.011 | 1.387 ± 0.024 | 2.877 ± 0.029 |
| SFE22 | 0.056 ± 0.003 | 0.252 ± 0.015 | 0.046 ± 0.001 | 0.097 ± 0.001 | 0.304 ± 0.022 | 0.710 ± 0.019 | 1.465 ± 0.021 |
| SFE23 | 0.147 ± 0.004 | 0.629 ± 0.033 | 0.141 ± 0.007 | 0.245 ± 0.013 | 0.734 ± 0.026 | 1.741 ± 0.060 | 3.638 ± 0.090 |
| SFE24 | 0.052 ± 0.001 | 0.230 ± 0.013 | 0.041 ± 0.002 | 0.091 ± 0.003 | 0.283 ± 0.006 | 0.664 ± 0.052 | 1.359 ± 0.069 |
| SFE25 | 0.165 ± 0.012 | 0.704 ± 0.056 | 0.160 ± 0.014 | 0.274 ± 0.010 | 0.818 ± 0.036 | 1.986 ± 0.060 | 4.107 ± 0.020 |
| SFE26 | 0.078 ± 0.003 | 0.368 ± 0.013 | 0.062 ± 0.001 | 0.139 ± 0.009 | 0.447 ± 0.021 | 1.061 ± 0.038 | 2.155 ± 0.047 |
| SFE27 | 0.106 ± 0.003 | 0.446 ± 0.032 | 0.095 ± 0.003 | 0.178 ± 0.009 | 0.524 ± 0.024 | 1.225 ± 0.064 | 2.573 ± 0.067 |

Each sample was measured three times in parallel, and the values were expressed as mean ± standard deviation (SD) in each sample. EPA: eicosapentaenoic acid; ALA: α-linolenic acid; DHA: docosahexaenoic acid; ARA: arachidonic acid; LA: linoleic acid; OA: oleic acid.

**Table S3.** The correlation coefficient of Pearson correlation analysis

|  | EPA | ALA | DHA | ARA | LA | OA | Principal UFAs | RCOO |
| --- | --- | --- | --- | --- | --- | --- | --- | --- |
| EPA | 1.0000 | 0.9982 | 0.9851 | 0.9990 | 0.9966 | 0.9970 | 0.9983 | 0.9267 |
| ALA | 0.9982 | 1.0000 | 0.9784 | 0.9990 | 0.9989 | 0.9986 | 0.9994 | 0.9204 |
| DHA | 0.9851 | 0.9784 | 1.0000 | 0.9771 | 0.9706 | 0.9759 | 0.9782 | 0.9725 |
| ARA | 0.9990 | 0.9990 | 0.9771 | 1.0000 | 0.9991 | 0.9985 | 0.9993 | 0.9132 |
| LA | 0.9966 | 0.9989 | 0.9706 | 0.9991 | 1.0000 | 0.9991 | 0.9993 | 0.9065 |
| OA | 0.9970 | 0.9986 | 0.9759 | 0.9985 | 0.9991 | 1.0000 | 0.9997 | 0.9176 |
| Principal UFAs | 0.9983 | 0.9994 | 0.9782 | 0.9993 | 0.9993 | 0.9997 | 1.0000 | 0.9197 |
| RCOO | 0.9267 | 0.9204 | 0.9725 | 0.9132 | 0.9065 | 0.9176 | 0.9197 | 1.0000 |

EPA: eicosapentaenoic acid; ALA: α-linolenic acid; DHA: docosahexaenoic acid; ARA: arachidonic acid; LA: linoleic acid; OA: oleic acid; UFAs: unsaturated fatty acids; RCOO: *Rana chensinensis* ovum oil
